# Complete Genome of *Rhodococcus pyridinivorans* SB3094, a Methyl-Ethyl-Ketone-Degrading Bacterium Used for Bioaugmentation

**DOI:** 10.1128/genomeA.00525-14

**Published:** 2014-05-29

**Authors:** Morten S. Dueholm, Mads Albertsen, Seth D’Imperio, Vaibhav P. Tale, Derrick Lewis, Per Halkjær Nielsen, Jeppe Lund Nielsen

**Affiliations:** a Center for Microbial Communities, Department of Biotechnology, Chemistry, and Environmental Engineering, Aalborg University, Aalborg, Denmark; b Novozymes Biologicals, Salem, Virginia, USA

## Abstract

Here, we present the complete genome of *Rhodococcus pyridinivorans* SB3094, a methyl-ethyl-ketone (MEK)-degrading strain used for bioaugmentation relating to the treatment of wastewater contamination with petrochemical hydrocarbons. The genome highlights important features for bioaugmentation, including the genes involved in the degradation of MEK.

## GENOME ANNOUNCEMENT

Members of the genus *Rhodococcus* are diverse, Gram-positive, nonsporulating, nonmotile, aerobic bacteria recognized for their outstanding catabolic capacity and physiological versatility, which allow them to adapt to a wide range of ecological niches (1, 2). Strains of *Rhodococcus* are commonly used for biodegradation due to their catabolic repertoire, which include pathways for the degradation of many compounds that cannot be easily transformed by other organisms, as well as for their ability to withstand harsh environmental conditions (1, 3).

*Rhodococcus pyridinivorans* SB3094 is a component of BioRemove 2300, a bioaugmentation product made by Novozymes Biologicals (Salem, VA) for enhanced hydrocarbon biodegradation in refining and petrochemical wastewater systems. The strain was isolated from a diesel waste site (Salem, VA) based on its ability to grow on methyl-ethyl-ketone (MEK) as its sole carbon source. Taxonomic assignment to *R. pyridinivorans* was based on 16S rRNA gene nucleotide sequence analysis and on physiological and biochemical features (4).

Genomic DNA was isolated using the PowerMicrobial maxi DNA isolation kit (MO BIO, Carlsbad, CA). Paired-end and mate-pair libraries were prepared with the Nextera DNA and mate-pair sample preparation kits (Illumina, Germany), respectively. The mate-pair library was prepared without any size selection. All procedures were carried out as recommended by the manufacturer. Sequencing of the paired-end and mate-pair libraries was performed using a HiSeq 2000 and MiSeq sequencer (Illumina, Germany), respectively. The reads were trimmed for adapters and quality and assembled *de novo* using the built-in tool of CLC Genomics Workbench v.6.0. The average coverage of the assembly was 249×. Manual scaffolding of the contigs was carried out based on paired-end and mate-pair information. Cytoscape version 2.8.3 (5) and Circos (6) were used for visualization and manual inspection of the assemblies, as described elsewhere (7). The gaps were closed and subsequently validated by manual read mapping in CLC Genomics version 6.0. Annotation was done using the NCBI Prokaryotic Genome Automatic Annotation Pipeline (PGAAP) (8).

The complete genome of *R. pyridinivorans* SB3094 is composed of a circular chromosome of 5,227,080 bp, a large, linear, single-copy plasmid of 361,397 bp, and a small, circular, multicopy plasmid of 2,035 bp. The overall G+C content is 67.8%. This bioaugmentation strain is most closely related to *R. pyridinivorans* AK37, with which it shares 98.2% average nucleotide identity using BLAST (ANIb) (9, 10). Annotation by the NCBI PGAAP identified 5,158 coding sequences (CDS), as well as 12 rRNA (5S, 16S, or 23S) and 55 tRNA genes.

*Pseudomonas veronii* MEK700 is able to grow on MEK using proteins expressed by the *mekABR* locus (11). A manual inspection of the SB3094 genome allowed us to identify an operon containing genes with very high similarity to the *mekA* (Y013_12765, 99.9% nucleotide identity) and *mekB* (Y013_12760, 99.8% nucleotide identity) genes of *P. veronii* MEK700. *R. pyridinivorans* SB3094 may consequently use a similar pathway for MEK degradation.

### Nucleotide sequence accession numbers.

The whole-genome sequencing project has been deposited at GenBank under accession no. CP006996 to CP006998.
